# Barriers to Weight Loss Among Inner-City Adults Seeking Obesity Treatment at an Academic Weight Management Program

**DOI:** 10.7759/cureus.83719

**Published:** 2025-05-08

**Authors:** Lissette M Céspedes, Alexis M Driscoll, Ashani Shah, Dhvani Doshi

**Affiliations:** 1 Internal Medicine, Rutgers University New Jersey Medical School, Newark, USA

**Keywords:** focus group, obesity epidemic, qualitative research, urban populations, weight loss

## Abstract

Objective

Obesity is an epidemic in US inner-city minority communities, which experience disproportionately higher rates of obesity and its preventable complications. There is limited published literature regarding weight management programs, particularly among inner-city academic centers caring for underserved patients. The primary purpose of this study was to improve our understanding of the patient experience with obesity by exploring the weight management journey, including barriers and facilitators for weight loss in patients seeking medical treatment for obesity at an inner-city, academic weight management center.

Methods

Three focus groups were conducted using a semi-structured discussion guide and qualitative study design. Participants, 18 years and older, were recruited from a single inner-city weight management program. A total of 18 participants attended one of three total focus groups. An interview guide was designed and utilized to elicit the experience of inner-city adult patients in the areas of their personal weight journey, where these patients obtain information about nutrition and physical activity, and which interventions participants would find most helpful to achieve successful weight loss. Investigators identified recurring themes from transcripts and coded remarks to themes from within and across the focus groups.

Results

Family support and personal motivation were important themes contributing to weight loss, while cost and time were significant barriers. Participants reported that the internet was their main source of information about weight management, which was supported by advice given by their physician or nutritionist. The primary intervention that participants recommended was improved accountability through support groups as part of a weight management program.

Conclusions

The findings of our qualitative research study highlight specific aspects of weight management which most highly affect weight journeys, including factors such as family support, the need for accountability, and challenges such as food, time, and mental health stressors, in turn allowing providers to prioritize these aspects when treating and guiding patients in weight management programs. These findings also provide important information to the leadership of weight management programs to guide future interventions; our institution aims to incorporate peer-led support groups to enhance accountability and communication with providers.

## Introduction

Obesity is a complex chronic disease associated with comorbidities that increase the risk of preventable, premature death from cardiovascular conditions, type 2 diabetes, and malignancy [1]. Obesity rates in the United States (US) continue to climb and have reached epidemic proportions. The most recent prevalence maps from 2023 show that all states have an obesity prevalence greater than 20%, with an average nationwide prevalence of 40.3% during August 2021-August 2023 [2]. Obesity disproportionately affects certain groups because of genetic, behavioral, and sociocultural factors [3]. Non-Hispanic Black adults have the highest age-adjusted prevalence of obesity (49.9%), followed by Hispanic adults (45.6%), non-Hispanic White adults (41.4%), and non-Hispanic Asian adults (16.1%) [4].

While there are genetic and personal medical conditions contributing to obesity, the lived environment and behavioral factors play a role in obesity development [3]. Environmental factors contributing to increased risk of obesity include less access to healthy food, excess low-quality food choices, limited availability of spaces for physical activity, and exposure to increased stress [3]. Obesity prevalence is known to be higher with health inequities. One study demonstrated that higher levels of social disadvantages have a 50-70% increase in obesity prevalence [5]. Rates of food insecurity are higher in low-income households, Black- and Hispanic-headed households, and households with children [6]. Stress associated with lower socioeconomic status (SES) may increase the likelihood of obesity through both biological mechanisms, such as increased cortisol, and behavioral mechanisms, such as less awareness of food consumption and less physical activity [5,7].

In Essex County, NJ, where this study was conducted, rates of obesity are estimated at 32%, which is higher than the state average [8]. This population has a higher percentage of residents from underrepresented minorities and a lower percentage of residents who identify as non-Hispanic White. According to county statistics, 37.7% of residents identify as non-Hispanic Black, 24.6% of residents identify as Hispanic, and 29.5% of residents identify as non-Hispanic White [8]. The county also has one of the lowest health rankings in NJ. Negative Social Determinants of Health (SDOH) burden is high in Essex County which has a higher percentage of uninsured residents (at 11%, compared to the state average of 8%), lower rates of high school completion, higher rates of unemployment, and higher rates of severe housing cost burden [8].

Unfortunately, obesity as a disease remains both underrecognized and undertreated, especially for patients of color. Patients of color are less likely to be diagnosed with obesity [3]. Studies show that despite higher rates of obesity in medically underserved populations, bariatric surgery rates are lower; most patients who undergo bariatric surgery are white, privately insured, and of higher SES [9]. Similar findings were reported in another study, which concluded that bariatric surgical rates are lower in minority populations despite higher prevalence of obesity [10]. Moreover, they found that physicians were less likely to recommend surgery as a treatment option to minority patients.

A better understanding of potential factors that affect obesity and successful weight management interventions in minority and under-resourced communities is required. Previous studies regarding patients’ perceptions revealed that exercise, support, and willpower, rather than biological factors, were necessary for successful weight loss [11]. Studies evaluating adult patients’ perspectives regarding weight management in medically underserved communities remain limited. Further research is needed to understand influences leading to weight gain and perceived health needs.

The primary aim of this study is to explore the experience of inner-city adult patients seeking medical treatment for obesity at an academic weight management program in the following areas: personal journey of weight, as well as motivators and barriers for weight loss. The second aim of this study is to understand where patients obtain information about healthy weight, nutrition, and physical activity. The third aim of this study is to investigate which interventions participants would find most helpful to achieve successful weight loss. The results of this study will be used to create new interventions in our weight management program to help patients achieve meaningful and long-term weight loss.

This article was previously presented as a poster at the 2024 Meeting of the American College of Physicians of New Jersey on March 8th, 2024, and the 2023 New Jersey Medical School Department of Medicine Research Day on May 18, 2023.

## Materials and methods

This study used semi-structured focus group discussions to explore the experience of inner-city adults with obesity in relation to their weight journey, motivators and barriers for weight loss, access to weight management resources, and desired program interventions for weight loss success.

Study participants

A total of 18 participants were included across three semi-structured focus group discussions. Inclusion criteria included adults aged 18 years or older enrolled in the weight management program at an academic, tertiary, inner-city public hospital. Exclusion criteria included inability to speak English and cognitive inability to participate in the discussion. A total of 139 adult patients from the weight management program were invited to participate. Convenience sampling was used for recruitment. Recruitment was conducted via phone calls between April 2022 and August 2022. Phone calls were made by research team members. Patients were given a brief overview of the study and asked if they were interested in participating. The most common reasons for refusal to participate included childcare/work responsibilities or transportation. Multiple session dates were offered, and the focus group times were set to be convenient for the highest number of attendees. Focus groups were completed between May 2022 and August 2022. The Rutgers University Institutional Review Board approved the study (#Pro2021001787). Before the start of the focus group, each participant provided informed written consent to participate in the study and to audio-record the focus group interviews. All participants were given a $10 gift card to the supermarket and provided with a meal during the focus group as compensation.

Focus groups

The focus group interviews were conducted by two study members (L.C. and D.D.). Each focus group had five to six participants. Focus group discussions were held in a private conference room at the hospital to ensure participant confidentiality and comfort. Each session lasted 60 minutes. The research team members facilitated the discussion. An interview guide was created to provide structure to the focus group (Table 1). The interview guide was designed to elicit the following: (1) the experience of inner-city adult patients in the areas of their personal weight journey including motivators and barriers for weight loss; (2) where patients obtain information about healthy weight, nutrition, and physical activity; and (3) which interventions participants would find most helpful to achieve successful weight loss.

**Table 1:** Focus Group Question Guide, by Topic.

Topic	Primary Question(s)	Prompting Questions
Ice-breaker	Tell us about your weight journey and what made you interested in a professional weight loss program?	What motivates you to lose weight?
Personal perceptions on obesity/knowledge	What concerns, if any, do you have about your current weight or body image?	Has your healthcare provider ever discussed your weight with you or specifically recommended that you lose weight?
		Did any of your friends or family ever encourage you to be of a certain weight?
		Has the media played a role in what weight you think you should be?
Access to evidence-based obesity care	Think about times when you have tried to lose weight, where did you obtain advice about how best to lose weight?	Do you feel you could ask your doctors to help you to lose weight if you wanted to? Why or why not?
Barriers to healthy nutrition	What challenges do you face, if any, when trying to eat healthy?	What is your idea of a healthy meal?
		Do you feel like you generally know what types of food are considered healthy?
		Do you feel like you have access to healthy food in Newark?
		Is cost of food a factor?
		Is time for meal preparation a factor? Who generally prepares meals in your family?
Barriers to physical activity	What challenges do you face, if any, with following a regular exercise routine?	What activities do you consider to be exercise?
		What locations/options are in Newark if you wanted to get exercise?
		How much exercise do you feel is realistic for most people to do in a week?
Barriers and/or support for weight loss	What are your sources of motivation and support when trying to lose weight?	Do you feel you have support from your friends and family when you are trying to lose weight?
		Overall in your opinion, what is the biggest challenge that people face when they are trying to lose weight?
Future interventions	What would be the most helpful changes you would like to see in your community that would make it easier to follow a healthy lifestyle?	
	What do you feel would be the most helpful from your healthcare providers to help you lose weight?	

Data analysis

Each focus group session was audio-recorded. The recordings were transcribed verbatim by a transcription service. Following each focus group, two members of the research team independently reviewed each transcription to identify common and meaning-bearing themes within and across the focus groups (L.C. and D.D.). Coding was conducted manually without the use of qualitative data software. An iterative, inductive approach guided by thematic analysis principles for qualitative studies was used [12]. The coders independently reviewed each transcription for overlapping themes, and discrepancies were resolved through consensus. The research team reviewed the codes to identify overlapping themes and their frequency, and a final report of the themes identified from the interviews was generated. Three focus group sessions were conducted to ensure thematic saturation was reached.

## Results

Of the 18 participants interviewed, almost all the patients were women of ethnic minorities. The major categories discussed in the focus groups were journey of weight gain, motivators for weight loss, barriers for weight loss, support for weight loss, sources of information for weight management and healthy eating, what is considered healthy nutrition, barriers to healthy nutrition, what is considered exercise, barriers to physical activity, and interventions that would be helpful to include in a weight management program.

Regarding their personal history of weight gain, many participants reported having excess weight since an early age (Table 2). For example, one participant said, “My weight’s always fluctuated my whole life.” Depression and stress, as well as excess food intake, were also major contributors to their journey of weight gain. Weight gain during the COVID-19 pandemic also emerged as an important theme, with one participant sharing, “started gaining weight again around COVID time and it’s just been a struggle to get the weight back off” (Table 2).

**Table 2:** Participants' Personal Journey of Weight Gain: Notable Quotes by Theme.

Themes	N (%)	Notable Quote/Examples
Excess weight since early age	7 (39)	“I have been heavy my whole life pretty much”
		“I come from a big family that like to cook…I just got this fat gene”
Mental health as a contributor to excess weight	6 (33)	“So mine came from a combination of mental health, my kids, and then I had health problems”
Excess food intake	6 (33)	“I tried to moderate my eating. I’m going to try to eat healthy for 3-4 days”
COVID pandemic causing weight fluctuations	5 (28)	“Started gaining weight again around COVID time and it’s just been a struggle to get the weight back off”

Family and kids were the most common source of support for weight loss (Table 3), particularly the ability to engage more with family in physical ways, such as through play or being present for the family’s daily needs. Family was also identified as a major support for weight loss and a source for information about how to lose weight and what constitutes healthy nutrition (Table 3). Participants also reported that family support was important for progress toward weight loss. Participants stated, “I do have family support that helps a lot,” and “there is a lot of support in my house.” Family members were a frequent source of information for weight management and healthy eating (Table 4).

**Table 3:** Motivators and Support for Weight Management: Notable Quotes by Theme.

Topic	Themes	N (%)	Notable Quote/Examples
Motivators for losing weight	Improve overall health and well-being	7 (39)	“When you eat right, it keeps the youth on. You look good, you feel good, and then it keep them diseases down”
	Kids/family	6 (33)	“I want to run and play with my babies”
	Improve chronic pain	5 (28)	“Now that I’m getting old, I’m having a lot of arthritis pain and they said the weight, the joints is wearing down”
	Improve physical limitation	4 (22)	“And somebody else got to do something for you. I don't like people doing anything for me. I like doing my own thing”
Support for weight loss	Family	8 (44)	“I do have family support, that helps a lot”
	Insurance coverage	6 (33)	Patients having access to electronic benefit transfer (EBT) cards to cover cost of healthy groceries and/or protein shakes
	Friends/groups/accountability	5 (28)	“I have a friend that motivates me. We go to the house every Wednesday. We have a girl group. We do the exercise for 1 hour and then we have a women’s group like we sitting here talking a podcast”
	Self-motivation/consistency	3 (17)	“I had to be my biggest supporter. Because at the end of the day, I can’t do it for anybody else. I had to do it for me”
	Doctors	3 (17)	“They’re always helpful the doctors and they always have something to soothe you in terms of that and they tell you what to eat and ‘try this, try that’”

**Table 4:** Knowledge and Sources of Weight Management: Notable Quotes by Topic and Theme.

Topic	Themes	N (%)	Notable Quote/Examples
Sources of weight management and healthy eating	Social media/YouTube/internet	7 (39)	“The internet is so amazing”
	Physician-advised	6 (33)	“I went to the sugar doctor a couple of years ago and he told me to stop drinking pepsi, sugary drinks, bread and pasta and I lost 73 lbs”
	Nutritionist-advised	5 (28)	“The nutritionist recommend mad vegetables and protein, no starch”
	Family recommendations	4 (22)	“And certain things I already had some knowledge and background, the nutrition and my mom always made sure we had a balanced meal”
	Self-guided	3 (17)	“I had to go on my own speaking to other people, looking on the internet because there’s a lot of patients dealing with obesity”
What is considered healthy nutrition	Steamed/green vegetables	7 (39)	“In my house, if there was a vegetable on that plate, that was a healthy ass plate”
	Baking foods or air fryer	4 (22)	“Everything I do within my air fryer so that’s my healthy meal”
	Protein	3 (17)	“I always start with the meat and then if I'm still hungry, I'll go to the bread. By then, I'm too full to even hit the starch.”
	Low-carbohydrate/sugar-free options	3 (17)	“I buy sugar free ice cream”
What is considered exercise	Walking	10 (56)	“Walking the shitzu”
	Home exercises	9 (50)	Treadmill, stairs, push-ups, squats, chair exercises, video games
	Outdoor activities	3 (17)	“Branch brook park is a place where a lot of people go walking/jogging”
			Biking or rollerblading

The main barriers to healthy nutrition were cost and time (Table 5). Over 60% of participants reported cost as a barrier to healthy nutrition. One participant stated, “If you want to eat good and healthy, that’s expensive.” Meanwhile, other participants said, “Everything’s cheaper, that’s bad for you,” and “We’re in a food desert.” About 40% of participants recommended better insurance coverage for weight management programs, including for healthy foods (Table 6). Additionally, staying motivated and mental health were significant barriers to both weight loss and exercise. Seventeen percent of participants mentioned that mental health issues, such as stress, were a barrier to healthy nutrition because overeating or food was used as a coping mechanism. For example, one participant said, “Stressed back-to-back, so food was my getaway. No smoking, no drinking, nothing like that.”

**Table 5:** Barriers to Weight Management: Notable Quotes by Topic and Theme.

Topic	Themes	N (%)	Notable Quote/Examples
Barriers to weight loss	Staying motivated	7 (39)	“Like the drive, because I want to do it and I'll start off really strong. Day three, on day three, four, maybe even I might be good for a week. But then there's something and my motivation goes downhill”
	Mental health	6 (33)	“There’s a lot of trauma behind a lot of people’s reasons, I think”
			“I self-sabotage a lot, so that’s my problem. It’s not that there’s no support, I just get in my moods and I self-sabotage”
	Lack of support	5 (28)	“I don't try to tell a lot of people what's going on with me with this because everybody's always got their own opinion and it's never good opinion”
Barriers to healthy nutrition	Cost	11 (61)	“It’s expensive. If you want to eat good and healthy, that’s expensive”
			“Everything’s cheaper that’s bad for you. The budgets and the rising prices”
	Time	9 (50)	“I need somebody to cook it for me or whatever and just set it up for me. So that way I’m going to pack and I can grab it and go. Because my life is actually real busy”
			Food preparation
	Cultural norms	8 (47)	“Culture makes it hard because different cultures eat different things if you’re the only one in the house trying to eat different from everyone else, that’s one of the hardest challenges”
	Food taste	5 (28)	“I want to eat vegetables more, but I want to make it tasty”
	Lack of availability at local supermarket	5 (28)	“We’re in a food desert”
			“Everything is not accessible in our town. If I want to go to Amazon fresh, I’m going to get cheaper but there goes my gas”
	Mental health	3 (17)	“Stressed back-to-back. So food was my getaway, no smoking, no drinking nothing like that”
	Family food preferences	3 (17)	“When I send my sisters to the grocery shop for me, they don’t get what I have on the list. They get what they want”
Barriers to physical activity	Lack of motivation	4 (22)	“I have the gym stuff at home, I don’t even touch it”
	Pain	4 (22)	“I avoid the gym at all costs – my body hurt, my knees hurt, my back hurt”

**Table 6:** Future Interventions to Include in a Weight Management: Notable Quotes by Theme/Idea.

Themes	N (%)	Notable Quote/Examples
Accountability/support groups/coaches	8 (44)	“I think that it would be easier for everybody that needed to do the exercise if they have accountability coaches. So when you have somebody cheer you on or somebody working with you, if you don’t have sober mind yet, they’re there for you”
Demonstrations (healthy eating)	4 (22)	“The best thing to do is show people how"
		“Instead of ya’ll telling us how to eat show us how to eat”
Access to fresh produce	4 (22)	Farmers markets or food banks
Insurance benefit for weight management	4 (22)	“Sometimes it helps to change your health plan”
Mental health counseling	3 (17)	“The sleeve will help you not be so hungry but it doesn’t help what’s in your mind”
		“I do believe that finding a therapist and a psychologist that understand the weight journey and the weight situation”

Participants also identified cultural norms and family food preferences as a barrier to healthy nutrition, particularly if they were the only person in the household trying to follow a healthy nutrition plan (Table 5). One participant shared “When I sent my sisters to the grocery shop for me, they don’t get what I have on the list, they get what they want.” Most participants considered vegetables as an important component of healthy nutrition, followed by baking foods or using an air fryer. In fact, one participant said, “In my home, if there was a vegetable on that plate, that was a healthy plate” (Table 4).

Other study findings included that improving health was the most common motivation for weight loss (Table 3). One participant stated, “When you eat right, it keeps the youth on. You look good, you feel good, and then it keep them diseases down.” Improving physical mobility and reducing pain were also notable motivators (Table 3), while pain was also a barrier to physical activity (Table 5). Most participants (56%) reported walking as the primary activity they consider to be exercise, followed by home exercises (50%) (Table 4). Seventeen percent of participants identified outdoor activities such as biking and rollerblading as sources of exercise.

Participants reported the internet, social media, and YouTube as main sources of information about weight management and healthy eating (Table 4). This was followed by advice given by their physician or nutritionist. One participant shared, “They’re (physicians) always helpful, the doctors, and they always have something to soothe you in terms of that and they tell you what to eat and try this and try that” (Table 4).

The primary intervention participants recommended for inclusion in a weight management program was improved accountability through support groups or coaching (Table 6). A total of 44% of participants recommended accountability, such as a support group or a coach, as a valuable intervention for a weight management program. One participant stated, “a support group would really be good for people like us that struggle with our weight definitely cause this is like a counseling we got ourselves. We can share with each other, and you don’t have to feel bad about it” (Table 6). The second most recommended intervention, mentioned by 22% of participants, was having demonstrations, especially regarding healthy nutrition.

## Discussion

The importance of seeking patient perspectives to develop community-based interventions that address health disparities cannot be understated. This qualitative study aimed to understand the facilitators and barriers to weight loss among participants seeking treatment for obesity at our academic weight management program. These participants represented patients from an inner-city, medically underserved community facing significant health disparities. Thematic analysis revealed that the weight management journey of patients from communities with high negative SDOH burden and limited resources is affected by factors such as family support, the need for accountability, and structural challenges such as food, time, and mental health stressors. Participants expressed that coaching and support groups could improve accountability and outcomes of weight management programs.

Family support and influence were one among the most prominent themes across multiple categories. This finding has been noted in other qualitative research studies exploring facilitators for weight change in urban communities [13,14]. Similarly, a study evaluating the influence of social networks on patient attitudes found that family support has been shown to influence self-care behavior in patients with diabetes, including factors such as adherence to physical activity, nutrition, and medication, and blood sugar monitoring [15]. Conversely, participants noted that family food preferences could be a significant barrier to weight loss. A similar paradox was mentioned in another study in which participants of a small qualitative study reported family networks to be both a positive and a negative influence [16].

Not surprisingly, the primary barriers to healthy nutrition were cost and time, consistent with what is frequently reported in the literature [13]. Time, such as that needed to prepare healthy meals, was reported as a barrier by 50% of the participants. Staying motivated and maintaining mental health were also found to be significant barriers to weight loss. Stress-eating as a barrier to weight loss has been noted in several studies, including a qualitative study evaluating barriers to healthy eating in urban women, which is consistent with our patient population [17]. These findings support the fact that food insecurity, including limited access to healthy foods in low-income, minority neighborhoods, is related to obesity [6]. An interesting finding in our results was that safety, often noted as a barrier to physical activity in urban neighborhoods, was not mentioned in any focus groups as a barrier to physical activity [18]. This finding should be further explored.

The physician-patient relationship was a recurring theme among the participants. Participants identified physicians as a source of information for weight management, healthy eating, and support for weight loss. One study demonstrated that the primary care physician (PCP) was a source of support for weight loss among most participants [19], and another study found that a supportive patient-physician relationship is critical to patient success with weight loss [20]. The finding of physician support among our patients was reassuring since physician distrust has been linked to poor health outcomes in minority communities [21].

Consistent with repeated mentions of family and motivation, as well as the physician-patient relationship, the primary intervention participants recommended for inclusion in a weight management program was improved accountability through support groups or coaching. This is no surprise since lack of motivation, lack of support, and mental health issues such as stress and depression were primary themes regarding their personal weight journey. A recent qualitative study, which aimed to evaluate motivators and barriers to weight loss in patients with obesity, also reported accountability as a desired inclusion for a weight loss program [22].

The findings of our qualitative research study add to the body of research on weight management interventions for underserved communities, since studies of this kind are limited [14]. Our findings highlight specific aspects of weight management that highly affect weight journeys, allowing providers to prioritize these aspects when treating and guiding patients in weight management programs. These findings also provide important information to the leadership of weight management programs to guide future interventions; our institution aims to incorporate peer-led support groups to enhance accountability and communication with providers. Future directions include implementing support groups and coaching teams to improve accountability of patients, as recommended by focus group participants, and then assessing the impact of these interventions.

This study is not without limitations. First, the small sample size (n=18) limits the generalizability of our results. A broader participant pool may have revealed more perspectives. Furthermore, the participation rate was low, as only 18 participants agreed to 139 invited, which can lead to non-response bias. Additionally, individual demographic information such as age, race/ethnicity, SES, and co-morbidities was not systematically collected, which limits our analysis of confounding factors. While we noted that the majority of participants were women of ethnic minorities, a demographic group which may show different motivations, barriers, and recommended treatment support tools than a focus group of other demographic characteristics, we are unable to determine how these characteristics influenced the emerging themes. The potential for selection bias exists within our recruitment strategy, as participants were only recruited from an academic weight management program where patients are self-seeking treatment for obesity. Strengths of the study include the qualitative, semi-structured discussion focus group design, which allowed for extensive discussion. This reinforced our patient-centered approach to obtaining perspectives and insights from patients with obesity receiving care at the weight management center.

## Conclusions

The findings of our qualitative research study highlight specific aspects of weight management which most highly affect weight journeys, including factors such as family support, the need for accountability, and challenges such as food, time, and mental health stressors, in turn allowing providers to prioritize these aspects when treating and guiding patients in weight management programs. These findings also provide important information to the leadership of weight management programs to guide future interventions; our institution aims to incorporate peer-led support groups to enhance accountability and communication with providers.
